# Supplementary material for: Promoting energy conservation through attention control and construct activation: A field test at a campus laundry
Source: PLoS One. 2026 Apr 8;21(4):e0344049. doi: 10.1371/journal.pone.0344049 (PMC13061205; doi:10.1371/journal.pone.0344049)
Supplement: Attachment — Submitted filename: Review.docx [file pone.0344049.s001.docx]

**Review**

I commend the authors for addressing an important, practical issue. The increase in cold water use from 25% to 57% shows the impact of applied psychological science. The use of direct observation in both studies adds rigor to pro-environmental research. These findings are a valuable and scalable contribution.

**Key Comments for Improvement**

1. Theoretical Mechanism (Attention): The main weakness is that self-reported attention did not explain the intervention’s effect, even though the paper focuses on “Attentional Control.” You should discuss whether attention was just needed for noticing the messages, or if measurement issues hid a real effect. Please clarify why attention mattered but didn’t mediate the effect.

2. Study 2 Control Group Contamination: Not removing pre-existing signage in the control group threatens the study’s validity. This may have influenced the control group’s beliefs or behavior. Please discuss how this condition differs from a true baseline with no signage.

3. Low Normative Beliefs Scale Reliability: The reliability of the Normative Beliefs scale (α = .62) in Study 1 is low. Since the intervention used a strong normative message, this weak measurement may explain why you didn’t find an effect. Please note this as a limitation.

4. Limited Sample Generalizability: Over 90% of participants in both studies were first-year undergraduates, who have little laundry experience. State clearly that these results mainly apply to novice groups.

5. Need to Separate Intervention Elements: Future research should break down the intervention package (arrow + three messages) to see if just the arrow or just the control belief message is effective, making the intervention more efficient.

**Minor Grammar and Clarity Issues:**

page 11, line 233: Change to “The second was the self-reported question: ‘Which wash cycle did you select?’”

Page 10, line 217: Change “cutout window” to “large viewing window” or “large pane-glass window.”

Best regards

**Aftab Haider**

*Southwest University of Political Science and Law, China.*
