# Supplementary material for: Promoting energy conservation through attention control and construct activation: A field test at a campus laundry
Source: PLoS One. 2026 Apr 8;21(4):e0344049. doi: 10.1371/journal.pone.0344049 (PMC13061205; doi:10.1371/journal.pone.0344049)
Supplement: Attachment — Submitted filename: ACCA Response to Reviewers.docx [file pone.0344049.s003.docx]

Steven G. Buzinski, Ph.D.

Professor

Department of Psychology & Neuroscience

University of North Carolina

334 Davie Hall | CB #3270

Chapel Hill, NC 27599-3270

01 February 2026

Dear Dr. Afzal,

I am pleased to submit the manuscript entitled, “Promoting Energy Conservation through Attention Control and Construct Activation: A Field Test at Campus Laundry” for consideration for publication in *PLOS One*. It is 6,431 words. I submit it on behalf of Tatum A. Jolink, Olivia Smith, Amit Ariely, Paschal Sheeran, and myself. This manuscript is a revised version of an earlier manuscript submitted to *PLOS One* titled, “Promoting Energy Conservation through Attentional Control and Construct Activation: A Field Test at Campus Laundry” (PONE-D-25-45725). The work does not constitute a conflict of interest for any of the authors.

We are very grateful for your invitation to resubmit and greatly appreciate the detailed comments and feedback that we received. We have revised the manuscript to address these comments. Most broadly, we have attempted to clarify the contribution of our proposed theoretical mechanisms (attention and construct activation) and the limitations of the work. We have provided our responses to each reviewer comment, and additional journal requirements, below.

Thank you very much for your consideration and for the helpful comments. We believe that the manuscript is much improved because of them, and hope that you enjoy reading it.

Correspondence can be directed to Steven Buzinski (buzinski@email.unc.edu). Thank you.

Sincerely,

Steven G. Buzinski, Ph.D.

Response to Additional Requirements:

1. Please ensure that your manuscript meets PLOS ONE's style requirements, including those for file naming. The PLOS ONE style templates can be found at:

https://journals.plos.org/plosone/s/file?id=wjVg/PLOSOne_formatting_sample_main_body.pdf

**We have followed the PLOS ONE style requirements, and the manuscript is now in compliance.**

2. Please note that your Data Availability Statement is currently missing the repository name and/or the DOI/accession number of each dataset. If your manuscript is accepted for publication, you will be asked to provide these details on a very short timeline. We therefore suggest that you provide this information now, though we will not hold up the peer review process if you are unable.

**Repository name on OSF: Promoting Energy Conservation through Attention Control and Construct Activation: A Field Test at a Campus Laundry**

**Project DOI:** [**https://doi.org/10.17605/OSF.IO/BFYWK**](https://doi.org/10.17605/OSF.IO/BFYWK)

**Study 1 DOI:** [**https://osf.io/bfywk/files/osfstorage/604fc0e736e58b02a6bc24ea**](https://osf.io/bfywk/files/osfstorage/604fc0e736e58b02a6bc24ea)

**Study 2 DOI:** [**https://osf.io/bfywk/files/osfstorage/604fc6385ca6c700097880ea**](https://osf.io/bfywk/files/osfstorage/604fc6385ca6c700097880ea)

3. We note that Figure 1a in your submission contain copyrighted images. All PLOS content is published under the Creative Commons Attribution License (CC BY 4.0), which means that the manuscript, images, and Supporting Information files will be freely available online, and any third party is permitted to access, download, copy, distribute, and use these materials in any way, even commercially, with proper attribution. For more information, see our copyright guidelines:http://journals.plos.org/plosone/s/licenses-and-copyright. We require you to either (1) present written permission from the copyright holder to publish these figures specifically under the CC BY 4.0 license, or (2) remove the figures from your submission.

**Thank you for bringing this to our awareness. We have removed the copyrighted images.**

Response to Reviewers:

I commend the authors for addressing an important, practical issue. The increase in cold water use from 25% to 57% shows the impact of applied psychological science. The use of direct observation in both studies adds rigor to pro-environmental research. These findings are a valuable and scalable contribution.

**We thank the reviewer for the positive evaluation of the level of contribution of our research and their appreciation of the work’s translational value. We are grateful for the reviewer’s engaged and constructive feedback and have endeavored to address each of the points raised in the reviews.**

**Key Comments for Improvement**

1. Theoretical Mechanism (Attention): The main weakness is that self-reported attention did not explain the intervention’s effect, even though the paper focuses on “Attentional Control.” You should discuss whether attention was just needed for noticing the messages, or if measurement issues hid a real effect. Please clarify why attention mattered but didn’t mediate the effect.

**This is an important issue and we appreciate the reviewer’s request for greater clarity concerning the theoretical mechanism underlying the impact of our intervention. We have substantially revised the discussion to address the two points raised by the reviewer (pp. 20-21). First, we agree that a self-report measure of attention is suboptimal and that an ‘objective’ (i.e., device-based) measure would have afforded firmer conclusions about the role of attention. Second, the Attention-Readiness-Motivation framework indeed proposes that endogenous attention is a precursor of the impact of valuations but valuations are the proximal predictor of action. Our signage intervention simultaneously manipulated attention and valuation which makes it difficult to disentangle their effects. We point out that future studies should endeavor to manipulate attention and valuation in a factorial design to offer a stronger test of their individual and combined effects.**

2. Study 2 Control Group Contamination: Not removing pre-existing signage in the control group threatens the study’s validity. This may have influenced the control group’s beliefs or behavior. Please discuss how this condition differs from a true baseline with no signage.

**We should have been clearer about the nature of the control condition and thank the reviewer for bringing this issue to our attention. We have added a new paragraph to the method section in Study 2 (p. 15) and deleted reference to the control condition in the discussion. The new paragraph indicates that the control condition qualifies as a “treatment as usual” or “usual care” control and we reference Freedland et al.,’s (2019) seminal analysis of choice of intervention comparators. We point out that the laundry room signage required by the Housing Authority remained in place and no additional signage concerning cold-water laundering was provided. Thus, in our control condition, the laundry room was the same as it would have been if no intervention was tested, and was equivalent to the physical setting tested in Study 1.**

3. Low Normative Beliefs Scale Reliability: The reliability of the Normative Beliefs scale (α = .62) in Study 1 is low. Since the intervention used a strong normative message, this weak measurement may explain why you didn’t find an effect. Please note this as a limitation.

**We thank the Reviewer for this comment, as the lower scale reliability could indeed be one reason why normative beliefs were not correlated with experimental condition in Study 2 (*r*=.11). They were, we note, positively correlated with laundering with cold water, regardless of condition (*r*=.25). While speculative, another possibility for the lack of correlation between condition and normative beliefs is that the normative belief items focused on the importance or cleaning benefits of using cold water, whereas the normative message in the intervention was about fighting climate change. These different targeted normative beliefs may also explain the small correlation. We’ve noted the scale reliability as a limitation in the General Discussion on page 23.**

4. Limited Sample Generalizability: Over 90% of participants in both studies were first-year undergraduates, who have little laundry experience. State clearly that these results mainly apply to novice groups.

**Thank you for this comment. We have addressed it by adding the following language to page 23, “First, it may limit the generalizability of the results due to the demographics of the sample. A majority of the participants (94%) in the intervention study were first year university students. It is possible that this sample was relatively inexperienced in laundering, and thus our findings may be limited to novice groups.”**

5. Need to Separate Intervention Elements: Future research should break down the intervention package (arrow + three messages) to see if just the arrow or just the control belief message is effective, making the intervention more efficient.

**Thank you again. We agree with the reviewer and have addressed this point by adding the following language to page 23, “It was also the case that we were unable to decompose the influence of the two key components of our intervention – endogenous attention (i.e., the physical arrow and its placement) and psychological construct activation (i.e., the behavioral, normative, and control belief messages on the arrow). Factorial designs could be used in future research in order to formally test the relative, and interactive, influence of each.”**

**Minor Grammar and Clarity Issues:**

page 11, line 233: Change to “The second was the self-reported question: ‘Which wash cycle did you select?’”

**We have revised this line to reflect the reviewer’s language above.**

Page 10, line 217: Change “cutout window” to “large viewing window” or “large pane-glass window.”

**We have revised this line to, “large pane-glass window.”**
